# Association between children's resilience and practising oral habits: a cross-sectional study

**DOI:** 10.1038/s41415-023-5565-7

**Published:** 2023-02-17

**Authors:** Mennat A. A. Abd-Elsabour, Rasha M. Hatem Hanafy, Ola M. Omar

**Affiliations:** 41415184094001 Master´s Degree Student of Paediatric Dentistry and Dental Public Health, Faculty of Dentistry, Cairo University, Cairo, Egypt; Teaching Assistant, Paediatric and Community Dentistry Department, Faculty of Dentistry, Ahram Canadian University, Giza, Egypt; 41415184094002 Associate Professor of Paediatric Dentistry and Dental Public Health, Faculty of Dentistry, Cairo University, Cairo, Egypt; 41415184094003 Vice Dean for Postgraduate Studies and Research, Professor of Paediatric Dentistry and Dental Public Health, Faculty of Dentistry, Cairo University, Cairo, Egypt

## Abstract

**Introduction ** While resilience is the quality of being buffered against stressors, practising oral habits is suggested to be a maladaptive behaviour towards stressors. The relation between resilience and practising oral habits in children remains vague.

**Aims ** This study aims to investigate the association between practising oral habits and resilience in children aged 5-7 years.

**Materials and methods ** An electronic Google form questionnaire was distributed through social media among five schools' children's parents, utilising the Child and Youth Resilience Measure-Revised Person Most Knowledgeable version (PMK-CYRM-R) scale as a resilience assessment tool, and the third domain of interview part of the Nordic Orofacial Test-Screen (NOT-S) as a habit assessment tool. The questionnaire received 227 eligible responses which were divided into habit-free group (123; 54.19%) and habit-practising group (104; 45.81%). The third domain of the interview part of the NOT-S included sucking habit, bruxism and nail-biting habits. The mean PMK-CYRM-R scores were calculated for each group and statistical analysis was done using SPSS Statistics package.

**Results ** Total PMK-CYRM-R score was 46.05 ± 3.63 in the habit-free group and 44.10 ± 3.59 in the habit-practising group (p = 0.0001). Bruxism, nail-biting and sucking habit subgroups showed statistically significant lower personal resilience levels than the habit-free group.

**Conclusion **The results of the current study suggest that children with low resilience levels may be more likely to practise oral habits.

## Key points

- This research suggests an association between practising oral habits in children as a coping mechanism against usual daily stressors and their low level of psychological resilience, which is the ability to positively adapt to these stressors.
- Children practising oral habits are suggested to have lower resilience levels in terms of their personal qualities rather than their relationships with their caregivers.
- Highlighting the low level of psychological resilience in children practising oral habits could be beneficial in enrolling them in resilience training programmes as an early preventive measure, which will improve their overall quality of life and productivity as adults.

## Introduction

A habit is a goal-independent, cue-response association in the memory, acquired slowly through the repetition of an action in a stable circumstance, without intention.^1^ While stress is the perception that the required demands exceed the individual's available resources.^2^ Stress induces habit formation on a neurophysiologic level by selectively attenuating the intentional stimulus-driven response, which is known as deliberate action control, and promotes habit formation in vulnerable individuals.^3^ Zhou *et al*. confirmed in their study a significant correlation between early life stress and habitual formation.^4^ Practising habits could be beneficial in relieving anxiety and securing the individual's performance, but it also can be destructive in the long run, as in the case of practising oral habits.^1^

Oral habits were found to be associated with stress.^5^^,^^6^ Stressed individuals were found to have a higher probability of developing bruxism.^7^ Moreover, bruxism in school children was associated with childhood stress.^8^ Stress was reported to be a powerful stimulus in sucking habit persistence.^9^ Also, stress could be considered an aetiological factor of nail-biting*.*^10^ These oral habits often have a harmful, deforming effect on the dental and maxillofacial complex.^11^

Psychological resilience is 'protective factors which enhance a person's response to some adversitie'.^12^ The psychological resilience concept has two main cores: the severity of adversity being passed through and the positive adaptation of the individual to that challenge.^13^ The term adversity is any sort of misfortune, hardship, traumatic event, or difficulty that is incorporated into daily life.^14^ Individuals with high psychological resilience levels show a positive response to tense events and daily stressors or appraise it as a non-stressful situation.^13^ The psychological resilience study represents a paradigm shift from investigating the risk factors associated with psychiatric disorders, to identify the strength aspects that buffer another individual against such disorders.^13^

The exact association between low levels of psychological resilience and practising oral habits in children remains vague. Psychological resilience is relatively a recent variable that has not been adequately tackled in the dental field. This study hypothesised that children practising oral habits have lower psychological resilience levels and, thus, are less tolerant towards daily stressors, which will lead them to practice oral habits as a coping mechanism, compared to their peers who are habit-free. Therefore, this research aims to study the association between psychological resilience and practising oral habits in a group of Egyptian children aged 5-7 years.

## Subjects and methods

### Study design

This study was an analytical cross-sectional study, in which an electronic questionnaire, designed using Google forms, utilised the Child and Youth Resilience Measure-Revised Person Most Knowledgeable version(PMK-CYRM-R)^15^ as a measure of a child's psychological resilience, and the third domain of interview part of Nordic Orofacial Test-Screen (NOT-S),^16^ assessing the presence of oral habits and its subtype, was distributed on social media during the last week of January 2021.

### Ethical approval and trial registry

Ethical approval for the research protocol was obtained from Research Ethics Committee, Faculty of Dentistry, Cairo University, Egypt, approval number: 71120, approval date: 24/11/2020. This study was performed as per the ethical standards laid down in the Declaration of Helsinki. The trial was registered on ClinicalTrial.gov (ID: NCT04710511).

### Informed consent

The Google form started with an informed consent portion, contained a detailed explanation of the study protocol, and by clicking 'next', the parent agreed to participate in this study.

### Sample size calculation

Since no previous studies investigated resilience among the habit-free group in comparison to the habit-practising group, the sample size was initially estimated by using G* Power 3.1.9.7 software, according to the results of Kasparaviciene *et al*.,^17^ in which the prevalence of oral habits in children aged from aged 5-7 years was 16.9%, and by adopting a confidence interval of 95%, a margin of error of 5% with finite population correction, the predicted sample size was 216 participants. After completion of the study, the power of the study was calculated based on the effect size of the primary outcome 'total PMK-CYRM-R'. An effect size (d = 0.54) indicated an actual power of 98% with a significance level of 0.05 for the two-sided hypothesis test.

### Participants

A list of private schools in Cairo, Egypt, was obtained from the local educational administration. A toss between the schools was made so that every school had an equal chance to be included in the study. The five schools were randomly chosen according to the toss result. After the schools' approval, the questionnaire form link was distributed to the schools' social media groups, with the aid of the schools' administration personnel. The responses to the Google form were submitted to a Google account that was made specifically for the purpose of this study.

Inclusion criteria were children aged aged 5-7 years old and that their mothers received a high education degree (college or higher). Exclusion criteria were those suffering from any chronic medical conditions, previous hospitalisation, or loss of a close family member, such as a parent or a sibling, during the last year. Furthermore, children who reported practising more than one oral habit, or practising the habit for less than one year, were excluded from the habit-practising group.

The mother's low educational level, chronic medical conditions, previous traumatic events (such as the loss of a family member) or previous hospitalisation, were all potential confounders affecting the child's psychological resilience,^18^^,^^19^^,^^20^ and thus were restricted.

To minimise any potential reporting bias, all responses were considered, and any responses with missing, unclear, or conflicting information were not included in the analysis. Also, selection bias was omitted, as the participants self-selected themselves to fill out the questionnaire.

The participants were divided into a habit-practising group and a habit-free group based on the parents' answers to the third domain of the interview part of NOT-S,^16^ which was also utilised to subdivide the children of the habit-practising group into: bruxism, nail-biting and sucking habit subgroups.

### Data collection

After the informed consent, the Google form consisted of three sections. The first one was designed to obtain demographic information (child's age, sex, medical history, previous hospitalisation, previous loss of a close family member, such as parent or sibling in the last year, and the mother's educational level and work).

The second section consisted of the third domain of the interview part of NOT-S,^16^ with a check-box options design. The items used were 'the child bites their nails every day', 'the child sucks their fingers or any other object every day', 'the child bites their teeth together hard or grinds their teeth every day', and an additional option 'the child does not practice any of the above habits'. Also, there was a question about the time elapsed while the child is practising the habit if reported.

The third section utilised the PMK-CYRM-R,^15^ answered by the parent. The PMK-CYRM-R^15^ consisted of 17 items and each item was scored as three points for yes, two points for sometimes, or one point for no. Two other subscales were driven from the same 17 items: personal resilience, which reflected the perception of how liked the child is, getting peer support, social skills, the ability to meet educational standards, sense of harmony and being adjusted within the surrounding community, and the perception of being treated fairly; and caregiver resilience, reflecting the quality of the child-parent relationship.^15^

The PMK-CYRM-R has an overall score ranging from 17-51: the higher the score, the higher the resilience. To compare the personal resilience and the caregiver resilience, an 'ordinal to interval conversion table' was used to convert the two scales' scores into equally distributed values. Scores would range from 0 to 20 for both subscales.^15^

The PMK-CYRM-R and the third domain of the interview part of NOT-S were translated into Arabic using the standard method as recommended by *Beaton et al.*^21^ A pilot study was conducted by interviewing mothers to assess their acceptance and understanding of the items of the translated questionnaires.

### Statistical analysis

Statistical analysis was performed with SPSS Statistics 20, Graph Pad Prism and Microsoft Excel 2016. Data were explored for normality using Shapiro Wilk and Kolmogorov-Smirnov normality tests, which revealed that all data are parametric data (p >0.05). Qualitative data were presented as frequencies and percentages. Quantitative data were presented as means and standard deviation (SD) values. Comparisons between qualitative data were performed by chi-squared test, while quantitative data comparisons were performed using the students' t-test for comparison between two groups, and the one-way ANOVA test followed by Bonferroni's *post hoc* test for multiple comparisons. Statistical significance was established as p <0.05.

## Results

**Fig. 1:**
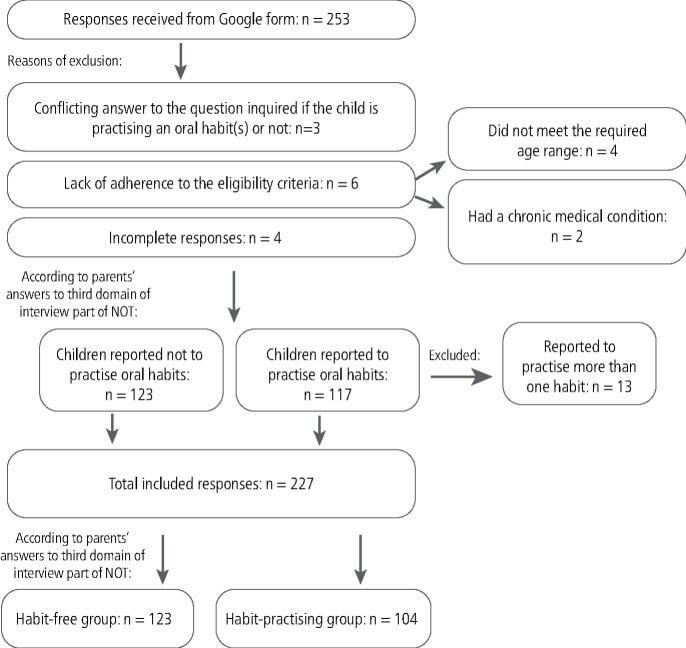
Flow diagram illustrating the number of the received responses, excluded responses, and included responses in the statistical analysis

The total number of the students aged from 5-7 years in the five schools was 347 children, and the received responses were 253, with a response rate of 72.9%. Excluded responses and the reasons for their exclusion are illustrated in Figure 1.

**Fig. 2:**
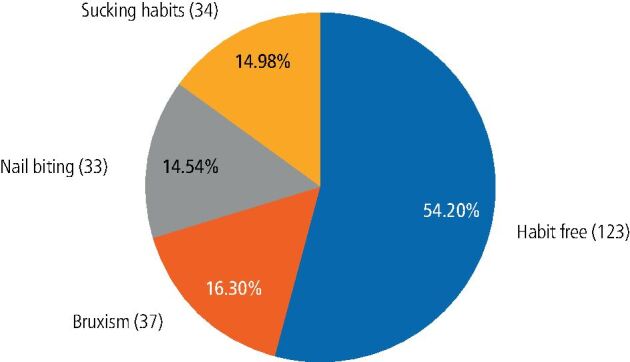
Pie chart illustrating the prevalence of different habits among the study population

A total of 227 responses were analysed. In total, 104 (45.81%) were practising oral habits, with a mean age of 5.82 ± 0.77; 58 (55.77%) of them were girls and 46 (44.23%) were boys. Additionally, 123 (54.19%) did not practice any oral habit, with a mean age 5.95 ± 0.86; 63 (51.22%) of them were girls and 60 (48.78%) were boys, without statistically significant difference between the mean age of the two groups, or the boy-girl distributions (p = 0.17, 0.54, respectively). The prevalence of each habit among the study population is demonstrated in Figure 2.

Table 1 shows the mean and standard deviation of resilience measures among the habit-free group in comparison to the habit-practising group. The mean and standard deviation of the resilience measures among the habit-free group and habit-practising subgroups showed in Table 2.

**Table 1:** Mean (M) and standard deviation (SD) of the resilience measures among the habit-free group in comparison to the habit-practising group (student t-test [two-tailed test])

Resilience measures	Habit-free group (n = 123)		Habit-practising group (n = 104)		P value
	M	SD	M	SD	
Personal resilience	15.49	2.66	14.07	2.15	0.0001*
Caregiver Resilience	18.11	2.24	17.27	2.47	0.0135*
Total PMK-CYRM-R	46.05	3.63	44.10	3.59	0.0001*
Key: * = statistically significant (p <0.05)

**Table 2:** Mean (M) and standard deviation (SD) of the psychological resilience measures among the habit-free group in comparison to the habit-practising subgroups (one-way ANOVA test with Bonferroni post hoc)

Resilience measures	Habit-free (n = 123)		Bruxism (n = 37)		Nail biting (n = 33)		Sucking habits (n = 34)		p-value
	M	SD	M	SD	M	SD	M	SD	
Personal resilience	15.49^a^	2.66	13.84^b^	2.76	14.15^b^	2.09	14.21^b^	1.67	0.0003*
Caregiver resilience	18.11^m^	2.24	17.62^n,m^	2.23	16.79^n^	2.80	17.53^m,n^	2.38	0.034*
Total PMK-CYRM-R	46.05^x^	3.63	44.03^y^	3.86	43.91^y^	4.06	44.41^x,y^	2.95	0.0013*
Key: * = statistically significant (p <0.05) Counts with the same superscript letters within the same comparison were insignificantly different (Bonferroni post hoc) Counts with different superscript letters within the same comparison were significantly different (Bonferroni post hoc)									

## Discussion

Resilience buffers the individual against life adversities. Children with low resilience are more fragile in the face of the usual daily stressors, which stimulate oral habit formation as a maladaptive behaviour.

The selected age range was 5-7 years to exclude the normal habit-practising age.^22^

Bruxism, nail biting and sucking habits were chosen, as the current evidence supports their association with psychological factors and disturbances.^22^^,^^23^^,^^24^

In this study, resilience was measured using PMK-CYRM-R as it is designed to be answered by the parent rather than the child, as the children in the selected age range were younger than being able to answer an online questionnaire by themselves, and their cognitive abilities might not be suitable to fully understand the items of the resilience measure well. In addition to being valid and reliable in diverse cultures, with adequate psychometric properties, PMK-CYRM-R investigates the child's resilience main components, personal resilience and caregiver resilience.^15^^,^^25^

Following the same methodology utilised by Leme *et al*.,^6^ the presence or absence of oral habit(s) and the type of habit if present were assessed using the third domain of NOT-S.^16^ NOT-S is a popular screening tool, proved to be valid and reliable in detecting orofacial dysfunction elements, such as practising oral habits, in participants aged three years or older. The domains of the interview part of NOT-S could be answered by the parents in the young age range.^16^

A Google form was easier to spread through social media, especially during the COVID-19 pandemic and ensured a random sample. The study sample was drawn from private schools, ensuring middle to high socioeconomic populations, which guaranteed the presence of basic life elements, excluding poverty as a possible confounder affecting the child's psychological resilience, as suggested by Gartland *et al*.^18^ Hirao* et al.*^26^ concluded that psychological online surveys measuring perception and impression had validity and reliability comparable to the surveys administrated in laboratory settings.

The mother's low educational level was suggested to be a risk factor associated with the child's oral habits.^20^ Moreover, a higher mother's educational level was found to be related to higher resilience levels in their children,^19^ which leads us to conclude that a low mother's educational level could be a possible confounder affecting the child's resilience. No evidence supports the influence of the father's educational level on either practising oral habits or psychological resilience.

The prevalence of oral habits in the study sample was 45.81%, which falls within the range previously reported in Egypt among school children aged from 6-12 years (67.9%) and the practice (29.1%).^27^^,^^28^

Although resilience scores in both study groups were relatively high, owed to the eligibility criteria in the recruited sample that omitted possible variables compromising the child's resilience,^18^ the total PMK-CYRM-R score in the habit-practising group was significantly lower than the habit-free group, which suggested that the children in the habit-practising group were more vulnerable, due to their own personal qualities, than their peers in the face of the stressors, driving them to practice oral habits as a coping mechanism. This conclusion is supported by the mean difference between the habit-practising and the habit-free groups regarding personal resilience was found to be higher than that of the caregiver resilience (Table 1).

Children in the habit-practising group scored a lower personal resilience score than in the habit-free group. These findings go in agreement with Leme* et al.,*^6^ who conducted their study on 147 students aged from 8-14 years, divided them into a habit group and habit-free group, and demonstrated the fragile personal qualities of the children and adolescents practising oral habits compared to their peers, in the form of high anxiety, assessed by the Revised Children's Manifest Anxiety Scale, (13.7 ± 5.6, and 16.1 ± 6.9, respectively), and depression, assessed by the Children's Depression Inventory level (7.5 ± 4.2, and 10.4 ± 7.0, respectively).

In this study, parents of children practising bruxism reported statistically significant lower mean resilience scores compared to the habit-free group. This result is consistent with the report of Oliviera *et al*.,^23^ who investigated the perception of parents of 84 bruxer children, aged 6-8 years, towards their children's personal qualities as being nervous, anxious, fearful, and aggressive, in comparison to the control group, and found that these qualities were more prevalent in the bruxer group (50%, 83.3%, 42.9%, and 16.7%, respectively) than in the control group (26.2%, 47.6%, 31%, and 4,8%, respectively), with statistically significant differences at being nervous and anxious items (p = 0.04, and p = 0.001, respectively).

Concerning the nail-biting habit, the finding of the current study follows Sisman* et al.,*^24^ who conducted their research on 724 school students aged 11-17 years, using the Brief Symptom Inventory scale, and concluded that the nail-biting group children scored higher anxiety (0.81 ± 0.72), depression (1.01 ± 0.88), negative self-image (0.87 ± 0.78), and hostility (1.18 ± 0.81), in comparison to their habit-free counterparts (0.55 ± 0.56, 0.70 ± 0.66, 0.55 ± 0.57, and 0.91 ± 0.74, respectively), with a statistically significant difference (p = 0.00).

Regarding sucking habits, the current findings match the old-fashioned assumption of Sigmund Freud, in his psychoanalytical theory that owed the persistence of a thumb-sucking habit after the age of three to the presence of psychological disturbance.^22^ This could be justified by the fact that sucking habits, unlike nail-biting or bruxism, provide the child with a sense of security, emotional satisfaction and sensual gratification,^29^ which will reduce anxiety. The present findings go in contrast with Vanderas* et al.* and Folayan who reported no association between anxiety and a thumb-sucking habit.^30^^,^^31^ This contrast can be owed to that both studies assessed anxiety as an outcome but not the stressors which the child had to face, while the current study measured resilience, which is the quality to be buffered against stressors. It is assumed that when the vulnerable child, who has a low resilience level, is subjected to a stressful circumstance, they will be disturbed and will try to minimise their anxiety by using sucking habits as a coping mechanism, which will make them feel secure rather than anxious.

Resilience could be acquired through well-designed social programmes. It's recommended to involve the children practising oral habits in resilience training programmes, as the current evidence supports the effect of these programmes in improving the child's ability to face different types of adversities, which will improve their quality of life and secure professional productivity, even as an adult.^32^ Also, further studies are recommended to investigate the effectiveness of resilience programmes, along with the usual interceptive methods, in stopping oral habits.

This study was limited by the lack of follow-up on the dynamic change in the habit clinical picture in correlation to the change in the resilience throughout the child's growth period. Also, being a cross-sectional study, it was not possible to establish the causality of the association between practising oral habits in children and low resilience level, and the degree to which the participants were stressed was not assessed by any of the available validated stress measurement tools. Yet, this study suggested a possible hypothesis that can aid in further researches.

## Conclusion

The applicability of this study's findings on other populations depends on many variables. Resilience is a dynamic term, which varies by many aspects: socioeconomic level, cultural standards, individual's own experiences in life, educational level, age and sex are all different variables that define an individual's resilience. Therefore, further studies on different populations are recommended, although the main hypothesis that children practising oral habits have a lower resilience level than their habit-free counterparts is expected to remain valid.

The results of the current study suggest that children with low resilience levels may be more likely to practice oral habits.
